# Supplementary material for: Phytaspase Is Capable of Detaching the Endoplasmic Reticulum Retrieval Signal from Tobacco Calreticulin-3
Source: Int J Mol Sci. 2023 Nov 20;24(22):16527. doi: 10.3390/ijms242216527 (PMC10671509; doi:10.3390/ijms242216527)
Supplement: Supplementary file 1 [file ijms-24-16527-s001.zip › ijms-2735666-supplementary.pdf]

Figure S1. Mass spectra of Lys-C peptides originated from **(a)** non-treated and **(b)** *Nt*Phyt-treated CRT3 bands (arrows in Figure 1A); **(c)** non-treated and **(d)** *Nt*Phyt-treated CRT3 D420E bands (lanes 3 and 4 in Figure 2A). Positions of the characteristic C-terminal semi-LysC peptides and of the N-terminal peptides are marked in red.

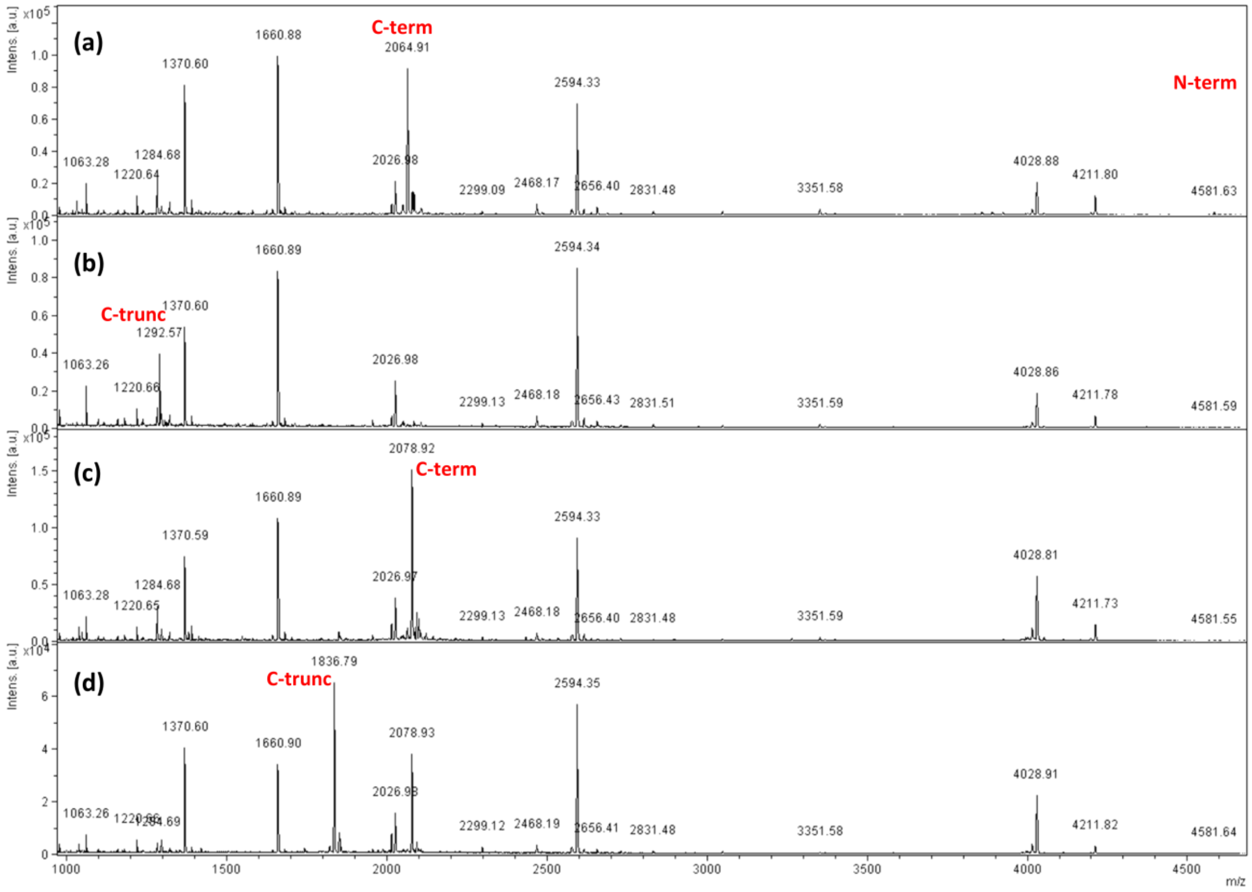

Figure S2. MS/MS fragmentation spectra of the characteristic C-terminal semi-LysC peptides of CRT3 (**(a)** non-treated, **(b)** *Nt*Phyt-treated) and CRT3 D420E mutant (**(c)** non-treated, **(d)** *Nt*Phyt-treated) marked in Figure S1.

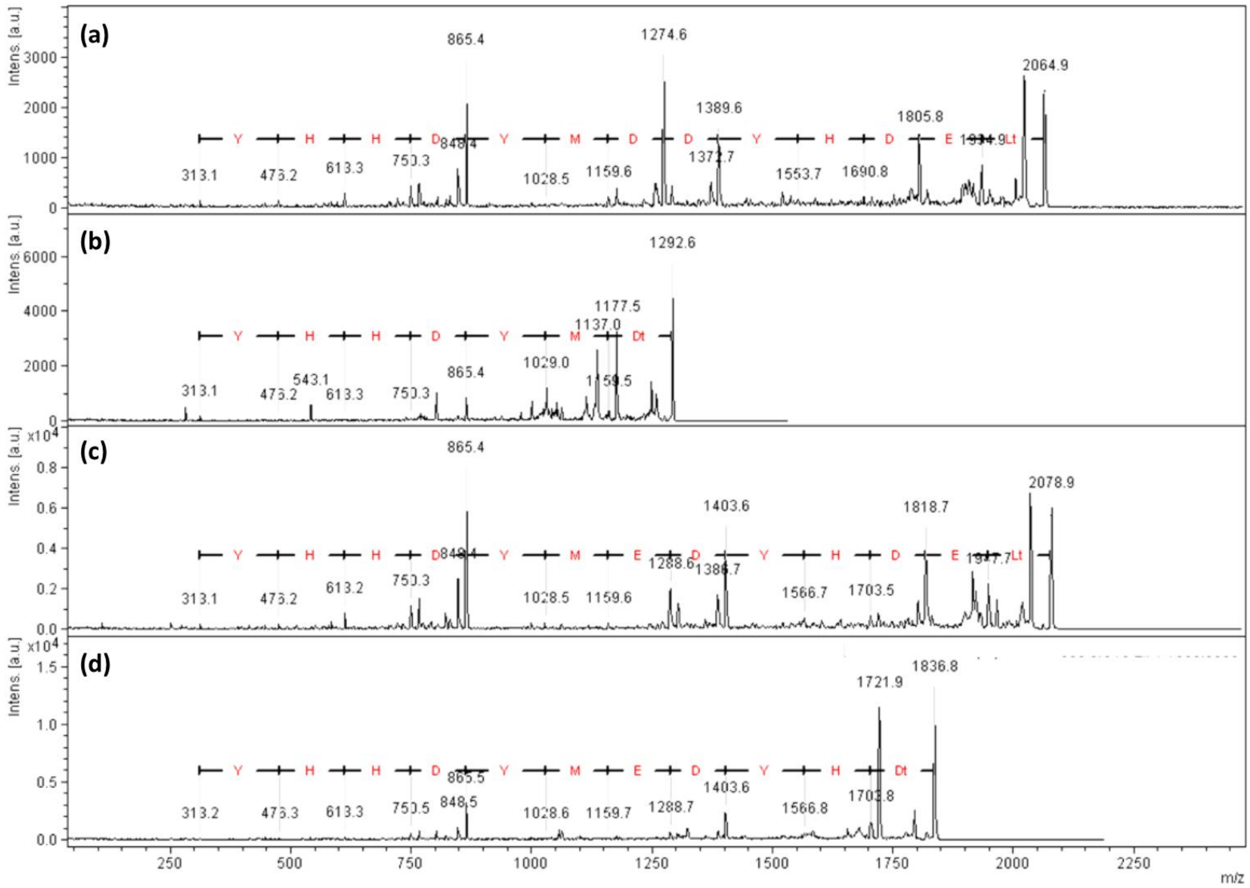

Table S1. List of the primers used in this study.

#	Primer name	Sequence
1	CRT3_Pst700_dir	AAGCCTGCAGACTGGGAAGAC
2	CRT3_D420E_Sac_rev	CGGAGCTCTTAAAGTTCATCATGGTAGTCTTCCATATAATC
3	CRT_Kpn_Nco_dir	CAAGGTACCATGGCTCTCTCTGAGCATAAAC
4	CRT_SP_Sal_rev	GTGGTCGACGAAAATCTCAGATGCTGAAG
5	CRT_LF_Apa_Nde_dir	GAGGGCCCGCATCTCATATGTCTGAGATTTTCTTTGAAGAAAG
6	CRT_1-420_Sac_rev	CGGAGCTCTTAATCCATATAATCGTGGTGATAGC
7	EGFP_Sal_dir	GAGGTCGACAAATCTGAGATGGTGAGCAAGGGCGAG
8	EGFP_Apa_rev	GTGGGCCCACCTGGAGACTTGTACAGCTCGTCCATGC
9	pLH_seq_dir	GACCTCGAGAATTCTCAAC
10	NtPhyt-His_rev	GGGAGCTCTTAATGGTGATGATGATGGTGCAGAGGATCCACTCCTATG
